# Cluster-Assisted Mesoplasma Chemical Vapor Deposition for Fast Epitaxial Growth of SiGe/Si Heterostructures: A Molecular Dynamics Simulation Study

**DOI:** 10.3390/ma17102448

**Published:** 2024-05-19

**Authors:** Wen-bo Wang, Wenfang Li, Ryoshi Ohta, Makoto Kambara

**Affiliations:** 1 School of Materials Science and Engineering, Dongguan University of Technology, Dongguan 523808, China; w-b.wang@hotmail.com; 2 School of Materials Science and Engineering, South China University of Technology, Guangzhou 510640, China; 3 Department of Materials Engineering, The University of Tokyo, 7-3-1, Hongo, Bunkyo, Tokyo 113-8656, Japan; 4 Department of Materials and Manufacturing Science, Osaka University, 2-1, Yamadaoka, Suita 565-0871, Japan

**Keywords:** SiGe alloyed films, atomic modeling, co-condensation, impingement, film growth

## Abstract

Co-condensation of mixed SiGe nanoclusters and impingement of SiGe nanoclusters on a Si substrate were applied using molecular dynamics (MD) simulation in this study to mimic the fast epitaxial growth of SiGe/Si heterostructures under mesoplasma chemical vapor deposition (CVD) conditions. The condensation dynamics and properties of the SiGe nanoclusters during the simulations were investigated first, and then the impingement of transient SiGe nanoclusters on both Si smooth and trench substrate surfaces under varying conditions was studied theoretically. The results show that the mixed nanoclusters as precursors demonstrate potential for enhancing epitaxial SiGe film growth at a high growth rate, owing to their loosely bound atomic structures and high mobility on the substrate surface. By varying cluster sizes and substrate temperatures, this study also reveals that smaller clusters and higher substrate temperatures contribute to faster structural ordering and smoother surface morphologies. Furthermore, the formed layers display a consistent SiGe composition, closely aligning with nominal values, and the cluster-assisted deposition method achieves the epitaxial bridging of heterostructures during cluster impingement, highlighting its additional distinctive characteristics. The implications of this work make it clear that the mechanism of fast alloyed epitaxial film growth by cluster-assisted mesoplasma CVD is critical for extending it as a versatile platform for synthesizing various epitaxial films.

## 1. Introduction

With the advancement of semiconductor technology, there is an increasing demand for improved growth, deposition, and processing techniques for epitaxial layers [1]. Although the electronic properties of the SiGe/Si heterostructures are not superior to those of third-generation semiconductors like SiC, GaN, AlGaAs, etc., they still attract a lot of attention due to their low-cost mass production in fabrication technology and being well suited to existing Si production lines [2]. For example, the Si modulation-doped field-effect transistor integrates well with Si-based technology [3]. However, the cost of industrial scaling is the high quality of epitaxial films, because the strained quantum well needs a thick epitaxial SiGe buffer layer to act as a virtual substrate. Low growth rates limit conventional growth techniques like ultrahigh vacuum-chemical vapor deposition (e.g., 0.13 nm/s for Si_0.8_Ge_0.2_ layers [4]), resulting in time-consuming processes and production defects.

In this regard, we have proposed that a cluster-assisted CVD process, termed mesoplasma CVD, provides a unique plasma environment for fast epitaxial Si film deposition at a rate of about 800 nm/s and a substrate temperature of 360 °C [5]. This high-rate epitaxial deposition is achieved owing to the nanoclusters formed as growth precursors when the fully decomposed source gas passes through the thermal boundary layer between the tail of the plasma and the substrate surface. We have confirmed the formation of Si clusters within the thermal boundary layer region ahead of the film growth surface experimentally [6]. Additionally, we simulated the dynamics of pure Si cluster formation and epitaxial Si film growth using MD simulations [7]. The results showed that the nanosized and loosely bonded clusters spreading instantaneously and spontaneously on the substrate’s surface contribute to fast epitaxial growth, i.e., the rapid atomic self-ordering of Si clusters, key to facilitating fast-rate epitaxy. Similar theoretical studies to identify the unique cluster-assisted growth of Si films have also been reported by other groups [8,9,10,11,12,13,14]. The mesoplasma CVD method has been proven to be useful for fast Si epitaxial film growth; however, its appropriability for binary SiGe films has not been explored.

From the perspective of traditional CVD technology, it is not difficult to transfer the conditions to binary systems by adding one more source material [15]. However, for the cluster-assisted mesoplasma CVD method, the result depends on the co-condensation of mixed nanoclusters and the fluctuation of cluster properties. The co-condensation of alloyed nanoclusters and the growth of heterostructures involve more parameters to tune, which should make the process more complicated than in a pure Si case. For example, when the additional thermodynamic variable, i.e., cluster composition, is considered, the cluster properties will vary depending on the different chemical activities of substances [16]. The mixed cluster’s formation is accompanied by free-energy change associated with the difference in the chemical potential of atoms, which may lead to surface enrichment or the segregation of the cluster structure [17]. Due to these features, more factors during deposition can influence the film growth of SiGe systems.

Computer simulation methods can provide detailed results, enabling an understanding of the growth process at an atomic level [18]. Therefore, in this work, MD simulations were applied to simulate the co-condensation of SiGe clusters and the impingement for epitaxial growth of alloyed SiGe heterostructures, both on smooth Si and trench Si substrate surfaces. The interface and surface morphology during regrowth, the redistribution of atoms, and the presence and nature of defects during growth were studied. Particularly, we learned the effects of substrate temperature, cluster size, and cluster composition on film quality. As a result, we demonstrated the successful modeling of epitaxial growth of binary SiGe films and show the great potential of the cluster-assisted mesoplasma CVD technique for the fast epitaxy of films in the semiconductor industry.

## 2. Simulation Method

MD simulations were performed by the commercial SCIGRESS (v2.4.1) program. The visualization and statistical analysis of the clusters were applied within OVITO (v2.9) [19], combined with a post-run Python (v3.6) script.

For cluster formation, the simulations were carried out in a cubic box with a cell length of 15 nm under the NVT (constant number, volume, and temperature) ensemble. We focused on the Si_0.5_Ge_0.5_ system using 1000 total target atoms (500 Si atoms and 500 Ge atoms) and added 1000 Ar atoms as a carrier gas to remove the latent heat during cluster formation. The initial configurations were constructed by the atoms distributed on the lattice site in the simulation cells at an appropriate distance to avoid the artificial initial colliding of the atoms. Initially, the cells were rapidly heated to 6000 K to achieve the vapor mixture state. Then, the atoms were cooled down to 1000 K at a rate of 2.7 × 10^12^ K/s to mimic the cooling process in real mesoplasma conditions. The condensed SiGe clusters denoted as Si_n_Ge_m_, then served as growth precursors in the subsequent cluster impingement simulations.

For impingement, the smooth surface of the substrate was set to a 32.6 × 32.6 Å area, and the trench surface was 38 × 27 × 33 Å. All these surfaces had five atomic layers of the Si (100) plane, for a total of 360 Si atoms. The bottom two planes were fixed, while the top three layers near the interface served as movable thermal-control layers. They were treated as an ideal heat reservoir to maintain a consistent substrate temperature. The atom velocities in the thermal layers were rescaled at each time step according to the set substrate temperature.

The force interactions used in the formation of SiGe clusters and impingement between the clusters and the substrate were modeled by the original Tersoff potential [20,21], which has been widely employed in Si, Ge, and C systems [22,23]. This potential, as a function of the atomic coordinates, is taken to be

(1) $$E=\sum_{i}E_{i}=\frac{1}{2}\sum_{i\neq j}V_{ij},V_{ij}=f_{C}\left(r_{ij}\right)\left[f_{R}\left(r_{ij}\right)+b_{ij}f_{A}\left(r_{ij}\right)\right],$$

(2) $$f_{R}\left(r_{ij}\right)=A_{ij}exp\left(-\lambda_{ij}r_{ij}\right),f_{A}\left(r_{ij}\right)=-B_{ij}exp\left(-\mu_{ij}r_{ij}\right),$$

(3) $$f_{C}\left(r_{ij}\right)=\left\{\begin{array}{l} 1,r_{ij}<R_{ij}\\ \frac{1}{2}+\frac{1}{2}cos\left[\pi \left(r_{ij}-R_{ij}\right)/\left(S_{ij}-R_{ij}\right)\right],R_{ij}<r_{ij}<S_{ij},\\ 0,r_{ij}>S_{ij}; \end{array}\right.$$

(4) $$b_{ij}=\chi_{ij}{\left(1+\beta_{i}^{n_{i}}\zeta_{ij}^{n_{i}}\right)}^{-1/2n_{i}},\zeta_{ij}=\sum_{k\neq i,j}f_{C}\left(r_{ik}\right)\omega_{ik}g\left(\theta_{ijk}\right),g\left(\theta_{ijk}\right)=1+c_{i}^{2}/d_{i}^{2}-c_{i}^{2}/\left[d_{i}^{2}+{\left(h_{i}-cos\theta_{ijk}\right)}^{2}\right]$$

(5) $$\lambda_{ij}=\left(\lambda_{i}+\lambda_{j}\right)/2,\mu_{ij}=\left(\mu_{i}+\mu_{j}\right)/2,A_{ij}={\left(A_{i}A_{j}\right)}^{1/2},B_{ij}={\left(B_{i}B_{j}\right)}^{1/2},R_{ij}={\left(R_{i}R_{j}\right)}^{1/2},S_{ij}={\left(S_{i}S_{j}\right)}^{1/2}$$

where *i*, *j*, and *k* are the atomic labels, *r_ij_* is the bond length between the atoms *i* and *j*, and *h_ijk_* is the bond angle between the *ij* and *ik* bonds. Here is a simple explanation of the physical meaning of each part: Equation (1) defines the total energy *E* of a system as the sum of pairwise potential energies *V_ij_* between particles *i* and *j*, and *V_ij_* is modified by a cutoff function *fc* that depends on their separation *r_ij_*. This potential is composed of repulsive *f_R_* and attractive *f_A_* terms, where the attractive term is modulated by a bond order term *b_ij_*. The functions of exponential repulsive and attractive forces are shown in Equation (2). The cutoff function *f_C_* in Equation (3) smoothly transitions the interaction from fully active to zero beyond a specified distance, avoiding abrupt changes which can cause numerical instability in the simulations. Equation (4) shows the function of bond order *b_ij_*, which adjusts the strength of the attractive interaction based on the local environment. Equation (5) computes average values for the parameters based on the properties of individual atoms or particles, allowing for a simple way to approximate interactions between different types of atoms in a mixed system. The parameters of the Si and Ge atoms we used can be found in reference [21]. Other interaction types (e.g., Ar-Ar, Ar-Si, Ar-Ge) were modeled by the Lennard-Jones potential [24]. The unlike-pair interactions of Ar-Si and Ar-Ge were calculated with the Lorentz–Berthelot mixing rules [25]. The potential cutoff distance was set to 15 Å for all the runs without tail corrections.

Although this potential has a shortcoming in that it overestimates the melting temperatures of pure Si and Ge [26,27], it provides accurate cross-potential parameters that could be used to model the binary Si-Ge system. In addition, the availability of nucleation and growth for mixed SiGe clusters has been verified by Nordlund et al. [28,29], and they have demonstrated that the Tersoff potential can effectively reproduce the structural properties of amorphous SiGe clusters. Thus, we considered it to be at least meaningful for describing the formation of amorphous SiGe clusters.

We used a velocity-scaling method to regulate the temperature of the systems [30]. We applied periodic boundary conditions to all the cell faces during cluster formation and to the four walls during impingement [31]. We set the time step for all the simulations to 0.5 fs. Furthermore, no additional impact energy was introduced on the clusters because a “soft landing” was considered in the actual experiment, with a velocity of several 10 m/s [32]. Therefore, the impinged cluster was set 10 Å above the top substrate surface and fell down onto the substrate with a kinetic energy of approximately 1.2 × 10^−3^ eV/atom. To avoid artificial interactions during the impingement of multiple clusters, we randomly selected the points of impact and sequentially impinged them at varying substrate temperatures.

## 3. Results and Discussion

### 3.1. Formation and Characteristics of Mixed SiGe Nanoclusters

**Figure 1:**
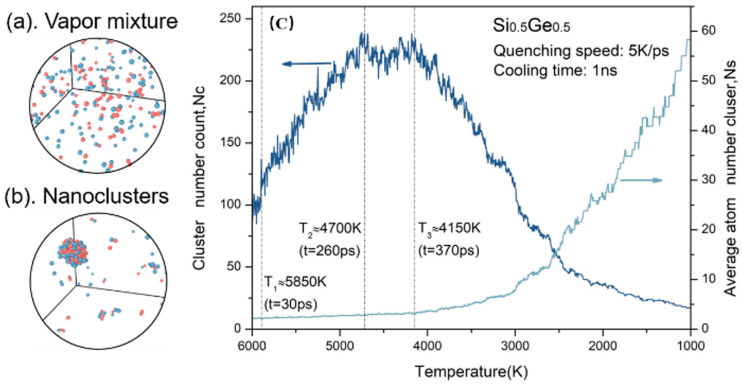
General course of the co-condensation process for the Si_0.5_Ge_0.5_ system with quenching. (**a**,**b**) snapshots of nanocluster formation in the cell at 5850 K and 2440 K. (**c**) The evolution of cluster number and average cluster size. The red dots are Si atoms and the bule dots are Ge atoms.

The simulation of cluster formation in the Si_0.5_Ge_0.5_ binary system was performed to mimic the growth precursors during cluster-assisted mesoplasma deposition. Figure 1 illustrates the general progression of cluster formation from vapor mixtures with quenching. Initially, at 5850 K, in Figure 1a, the Si and Ge atoms, in a vapor mixture state, show no significant cluster formation. Figure 1b shows clusters of various sizes at 2440 K as the cooling of the system, indicating the survival of large and stable clusters at this stage, corresponding to the temperature in the tail of the plasma. Statistically, Figure 1c illustrates the evolution of both cluster numbers and the average number of atoms at each temperature (i.e., average cluster size) during the co-condensation process. After a brief relaxation period before 30 ps, we observed a rapid increase in cluster numbers from T_1_ to T_2_, which then led to a quasi-equilibrium state where cluster formation and decay were in balance between T_2_ and T_3_. The profile of the average cluster size clearly shows that there are only small molecules at a high temperature, and the clusters primarily grow larger through coalescence at low temperatures as the monomers quickly deplete, as evidenced clearly by the average size increasing from 10 atoms to 60 atoms under 3000 K.

**Figure 2:**
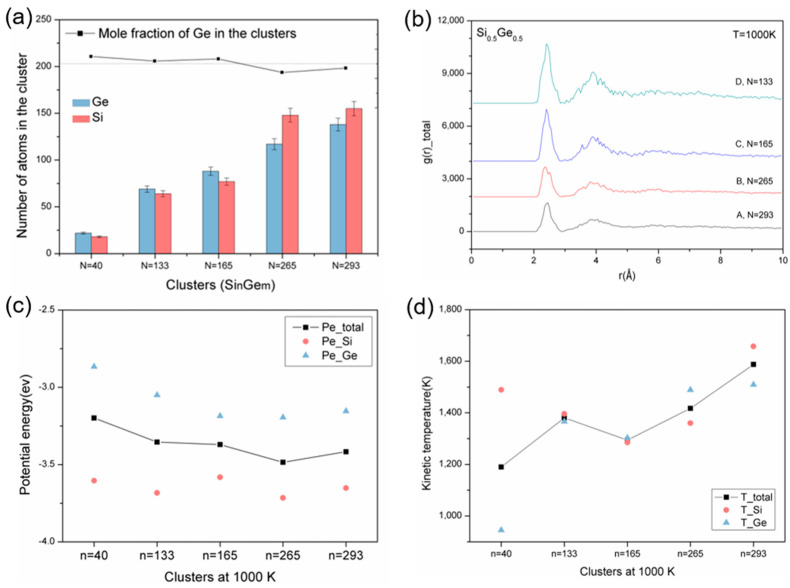
Properties of the SiGe mixed clusters at 1000 K: (**a**) Composition. The straight line is the nominal value of the mole fraction of Ge. (**b**) Structure. (**c**) Potential energy; and (**d**) Temperature.

To study the clusters used for impingement as precursors, we analyzed the final transient clusters observed at 1000 K for their compositions, structures, potential energies, and temperatures, as shown in Figure 2. Figure 2a clearly shows that the Ge mole fraction of all the selected clusters closely matches the nominal composition. Although there is a slight decrease in the Ge content as cluster size increases, the fluctuations remain minimal. This consistency indicates the reliable production of chemically uniform SiGe nanoclusters, independent of their size. Figure 2b displays the radial distribution function (RDF) of these clusters [33]. As cluster size decreases, increasingly sharp peaks at the first nearest neighbor reveal denser clusters, while the second neighbor peak indicates that all the clusters remain amorphous. As we know, liquid-like pure Si clusters spread and rearrange quickly on the substrate for epitaxial film growth. In the same way, mixed amorphous SiGe nanoclusters may also contribute to the fast epitaxial SiGe layer’s growth as precursors during deposition, which will be studied in detail in the next impingement section.

Figure 2c,d display the potential energies and temperatures of these clusters, including the average Si and Ge values. It can be seen that larger clusters exhibit a lower potential energy. On one hand, the surface atoms of a cluster experience fewer interactions compared to the internal atoms because they are not surrounded on all sides. As the cluster size increases, the proportion of internal atoms increases relative to the surface atoms. Consequently, larger clusters have more internal atoms, experiencing uniform attractive forces, reducing the high potential energy caused by surface effects. On the other hand, systems tend to evolve towards a state of minimum energy. In larger clusters, atoms have more space and opportunities to rearrange themselves to achieve lower energy states due to their more uniform internal energy distribution. Therefore, a higher proportion of internal atoms and thermodynamic stability may make larger clusters physically and chemically more stable and predisposed to formation. In addition, due to the higher potential energy of the surface atoms in a cluster, it is generally believed that the clusters should be slightly enriched with Ge atoms on the surface, which may additionally contribute to rapid spreading during impingement. Thus, compared to the pure Si case, a structured SiGe cluster, along with diverse cluster compositions, may yield unique dynamic behaviors during film growth. Due to insufficient heat dissipation [34], the temperature of the clusters, defined by the mean kinetic energy and derived from the average atom velocities, shows higher average temperatures than the system’s heat bath. As for the Si and Ge atoms, they exhibit irregular behavior due to the collisions of surface atoms with the surrounding argon atoms, depending on the different cluster sizes and compositions [35].

### 3.2. Impingement of a Single Nanocluster on the Smooth Si Surface

**Figure 3:**
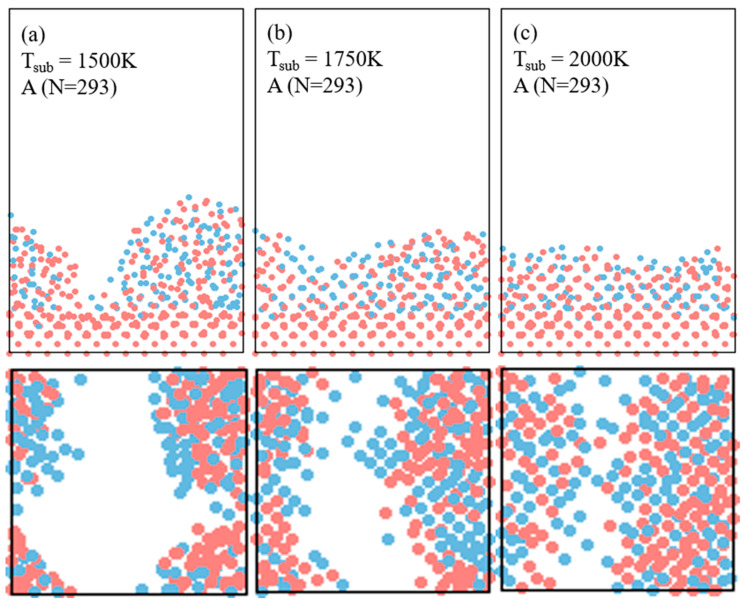
Snapshots of the single cluster of Si_155_Ge_138_ (A, N = 293) impinge on the Si smooth substrate at different temperatures: (**a**–**c**) substrate temperatures of 1500 K, 1750 K, and 2000 K, respectively. The red dots are Si atoms and the bule dots are Ge atoms.

Figure 3 illustrates the spreading and rearrangement of the single cluster of Si_155_Ge_138_ (noted as A, N = 293) on the smooth Si (100) surface at different substrate temperatures over a 5 ns interval. At 1500 K, as shown in Figure 3a, the cluster only spreads locally at the interface, maintaining most of its original globular shape. As the substrate temperature increases to 1750 K in Figure 3b, the cluster deforms extensively and spreads across the substrate surface in a planar shape. Although full monolayers of atomic planes are not present at the impact interface, the alignment of some atoms in the vicinity of the impact area to the epitaxial ordering structure can be observed. Particularly, from the top views shown in Figure 3a,b, it is evident that the Ge atoms diffuse faster and move over a longer distance compared to the Si atoms, which may correspond to the high potential energy of the Ge atoms on the cluster surface in Figure 2. When the substrate temperature reaches 2000 K in Figure 3c, the diffusion and rearrangement of both the Si and Ge atoms become pronounced due to the increased diffusion rates at higher temperatures. Utilizing a simple diffusion relation formula [36], $x=\sqrt{D}$, where *D* represents the diffusion rate, *t* the duration time, and *x* the distance of the atoms’ motion, it becomes clear that both the Si and Ge atoms can efficiently spread across the distance *x* to explore their energy-favorable positions, leading to a relatively uniform elemental distribution. Thus, substrate temperature significantly influences the degree of deformation and atomic self-ordering due to the substantial increase in the diffusion rate.

**Figure 4:**
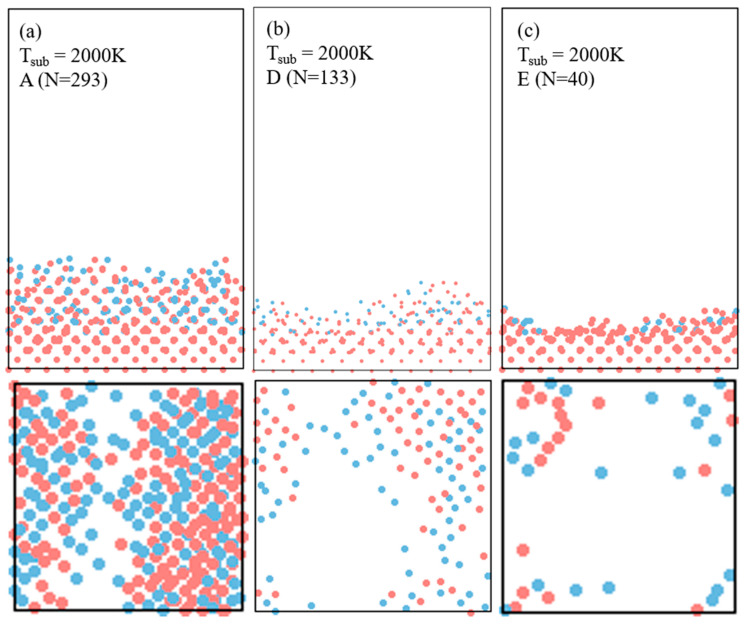
The effect of cluster size on epitaxial ordering layers on the Si (100) substrate at the substrate temperature of 2000 K. (**a**–**c**) Impingement by the clusters Si_155_Ge_138_ (A, N = 293), Si_69_Ge_64_ (D, N = 133), and Si_18_Ge_22_ (E, N = 40), respectively. The red dots are Si atoms and the bule dots are Ge atoms.

Figure 4 shows the reconstruction of atomic structures by the impingement of different-sized clusters of Si_155_Ge_138_ (A, N = 293), Si_69_Ge_64_ (D, N = 133), and Si_18_Ge_22_ (E, N = 40) on the substrate at 2000 K. The figures clearly show that all the clusters undergo complete deformation from their original globular shapes. However, as the cluster size decreases, it is apparent that the comprising atoms align more closely to the crystalline structure of the underlying Si (100) substrate. The ordering of the constituent atoms of the clusters is clearly visible for all the different-sized clusters, showing that the atoms are well aligned in the planes at uniform intervals at the interface. It should be noted that, although large clusters do not achieve as precise an ordering as smaller clusters, a rapid rate of epitaxy is expected due to the relatively large size of these growth precursors [7].

### 3.3. Impingement of Multiple Clusters on the Smooth Si Substrate

**Figure 5:**
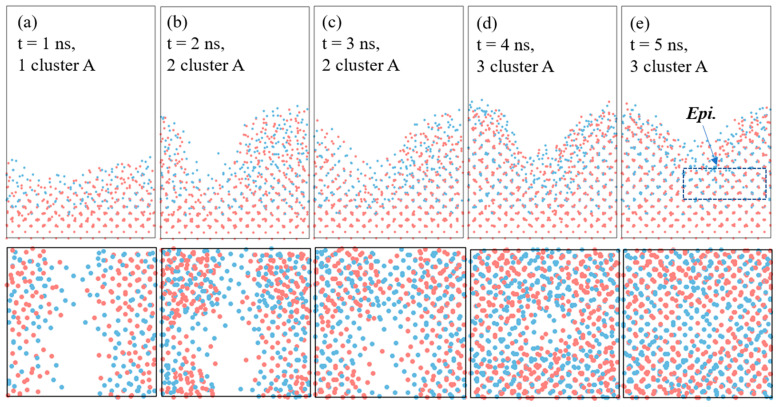
Evolution of deposition layers during the impingement of multiple clusters of Si_155_Ge_138_ (A, N = 293) on the Si (100) substrate at a velocity of 10 m/s for 5 ns at 2000 K. (**a**–**e**) Snapshots of interface and surface morphology at 1 ns, 2 ns, 3 ns, 4 ns, and 5 ns, respectively. The red dots are Si atoms and the bule dots are Ge atoms.

During the cluster-assisted deposition process, film growth is driven by the sequential impingement of numerous clusters, with earlier clusters spreading on the surface beforehand. To look into the atomic-level ordering of the SiGe/Si heterostructures, we simulated the impingement of three identical clusters at a substrate temperature of 2000 K for 5 ns on a smooth Si (100) substrate surface. As shown in Figure 5, we depicted the interface and surface morphology throughout the simulation period. Initially, the interface remained nearly flat for the first 1 ns in Figure 5a. Subsequently, as growth progressed, the interface became roughened, forming alternating crystalline planes. The simulation showed that the wavelength and height of the facets gradually decreased, indicating rapid spreading and diffusion at the growth front during solidification. Therefore, it could be verified that the unique aspect of liquid-like cluster deposition was that, following the spread of the initial cluster on the smooth Si surface, subsequent clusters landing on the roughened surface continued to deform and spread rapidly, forming local epitaxial layers. By the final growth stage, atoms had spread across the entire roughened surface, forming several epitaxial layers. The atoms near the interface aligned with the substrate at the base of the atomic facets.

**Figure 6:**
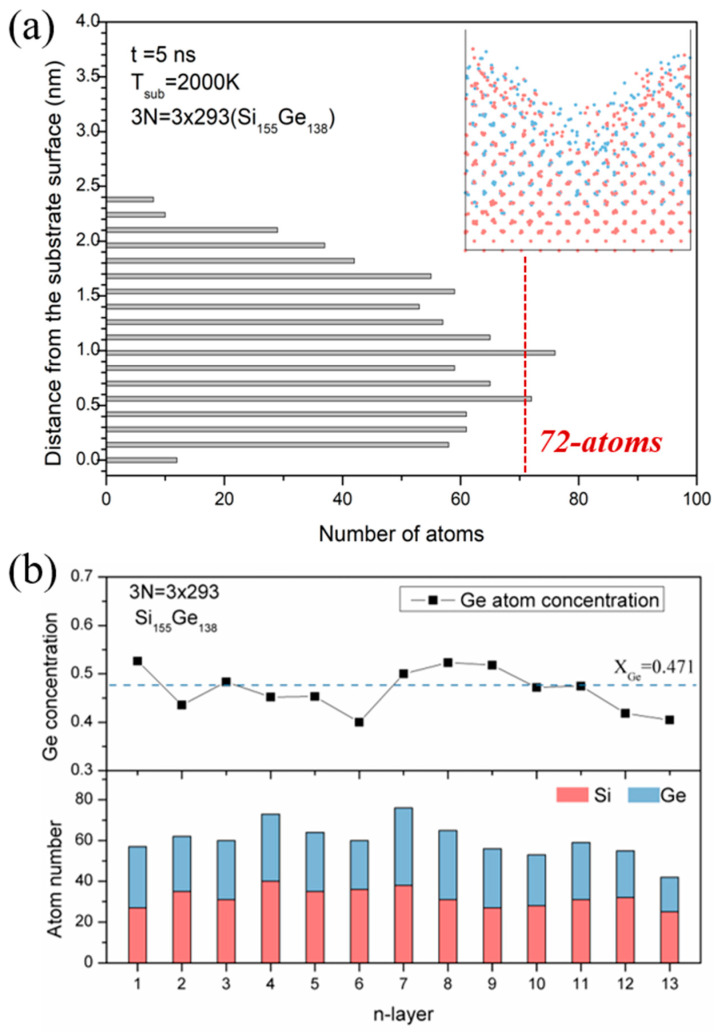
The elemental distributions of each layer, based on the structure of multi-cluster impingement on the smooth substrate at 5 ns. (**a**) The number of atoms counted from the substrate surface every 0.14 nm. (**b**) Histogram of Si and Ge numbers in each layer, as well as the Ge content. The red dots are Si atoms and the bule dots are Ge atoms.

To look into the composition of the layers, we analyzed the elemental distributions statistically at the atomic level, as shown in Figure 6. According to Vegard’s law, the lattice parameter of the Si_0.5_Ge_0.5_ alloy was 0.55 nm, and then the distance between the two neighboring atomic planes was calculated as 0.14 nm. In our study, we quantified the Si and Ge atoms in each plane from the interface by counting the number of atoms at increments of 0.14 nm for the final state at 5 ns (i.e., the inner image comes from Figure 5e). As shown in Figure 6a, most layers contained around 60 atoms, which corresponded to 83.3% of the full capacity of 72 atoms in one crystal facet. The self-ordering layers were about 1.8 nm thick and contained up to 13 atomic planes. A significant disordered region appeared beyond the 12th layer, with fewer than 40 atoms per layer, indicating an uneven surface structure. Therefore, this reveals that the continuous cluster deposition process allows for the rapid spreading of clusters to achieve the epitaxial structure, and we understand that the cluster-assisted mesoplasma CVD process can result in fast epitaxial ordering structures in the same way.

We also assessed the Ge concentrations in each layer by counting the Si and Ge atoms separately, as shown in Figure 6b. The nominal mole fraction of Ge in cluster A was around 0.471, which was slightly lower than the composition of the Si_0.5_Ge_0.5_ system. From the upper figure, it is clear that the mole fraction of Ge in each layer closely matches the nominal fraction, indicating chemical uniformity among the ordered atomic planes. Thus, we consider that precisely controlling the film’s composition during cluster-assisted CVD processes can be achieved by simply adjusting the source gas ratio, regardless of dependencies on the deposition parameters.

**Figure 7:**
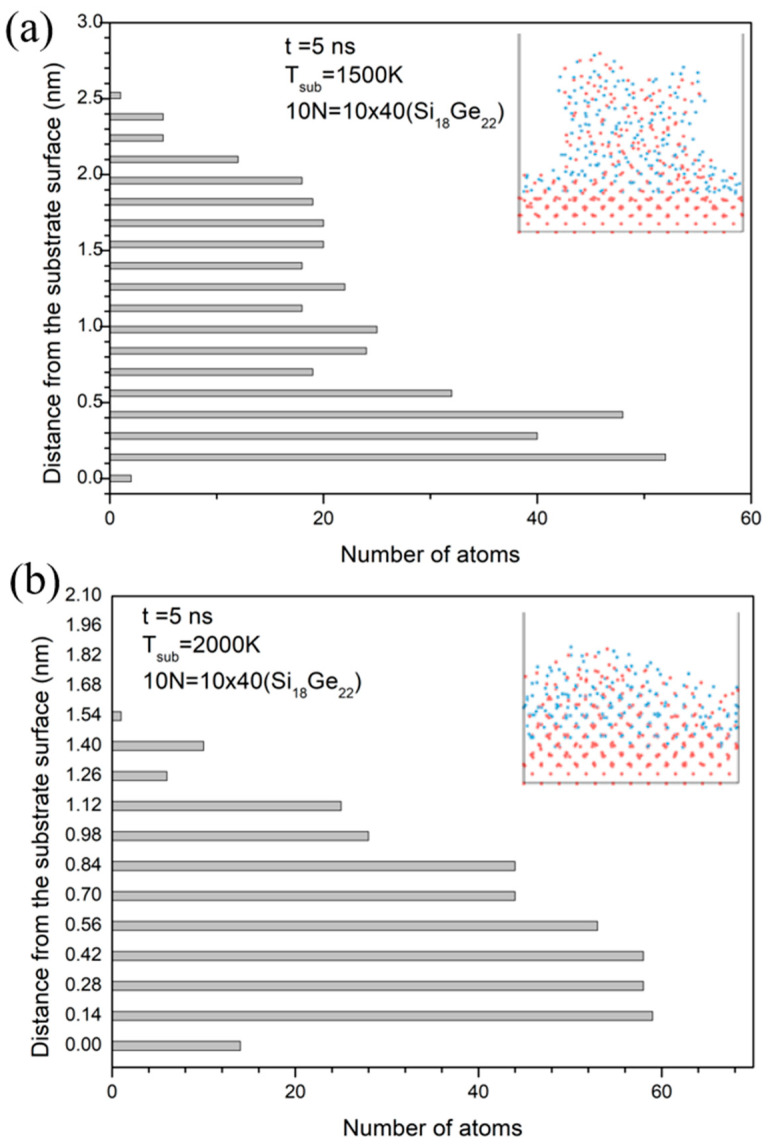
Effect of substrate temperature on the film structure by the impingement of a small multi-cluster of Si_18_Ge_22_ (E, N = 40). (**a**) The number of atoms in each layer and the snapshot of the morphology in the case of 1500 K. (**b**) The same as (**a**), but in the case of a substrate temperature of 2000 K. The red dots are Si atoms and the bule dots are Ge atoms.

To investigate the effects of substrate temperature on layer structure, we simulated the impingement of ten small clusters of Si_18_Ge_22_ (E, N = 40) under the same conditions as the impingement of cluster A. Using the same counting method as Figure 6, we briefly show the interface morphology and number of atoms in each layer in Figure 7. At a reduced temperature of 1500 K, the deposition forms mound-like island structures with a height of 2.5 nm, as shown in Figure 7a. It is clear that the number of atoms per layer decreases sharply, suggesting that clusters cannot sufficiently deform and spread at lower substrate temperatures. In contrast, at 2000 K, in Figure 7b, atoms distribute uniformly and align along the [100] direction, with no notable peaks in the atom count per layer, indicating significant cluster deformation and spreading. Moreover, compared to the case in Figure 6, films formed by smaller clusters have a smoother surface. These results demonstrate that substrate temperature and cluster size significantly influence both the degree of cluster spreading and the quality of films.

### 3.4. Bridging Growth by Multi-Cluster Impingement on Si Trench Surface

**Figure 8:**
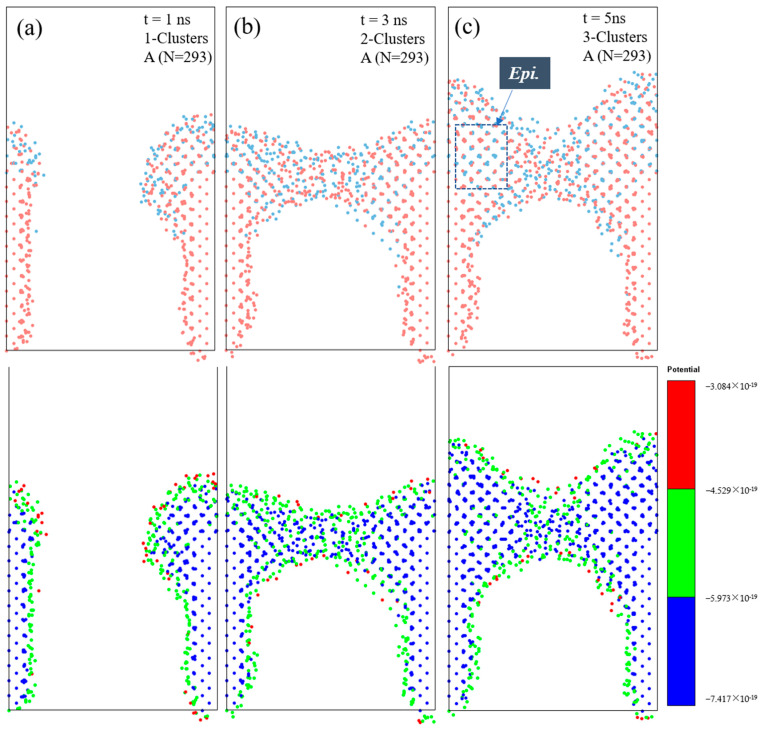
Evolution of morphology and potential energy for the impingement of multiple clusters of Si_155_Ge_138_ (A, N = 293) on a Si trench surface at a velocity of 10 m/s for 5 ns at 2000 K. (**a**–**c**) show snapshots of the bridging structure and the corresponding potential energy of all the atoms at 1 ns, 3 ns, and 5 ns, respectively. The red dots are Si atoms and the bule dots are Ge atoms.

To elucidate the distinctive features of cluster-assisted deposition, we conducted impingement simulations of multiple clusters of Si_155_Ge_138_ (A, N = 293) on a Si trench surface. Figure 8 shows the evolution of the structure and potential energy of the deposition layers during the impingement. Initially, at 1 ns, in Figure 8a, the first cluster adheres to the substrate’s wall near the surface, with a few ordered atoms exhibiting low potential energies. Then, by 3 ns, as shown in Figure 8b, the attachment of another cluster completes the formation of the bridging layers. It is clear to see that the atoms near the substrate wall solidify more rapidly than others. As depicted in Figure 8c, at 5 ns, the disordered region in the center of the bridging layers gradually aligns to the [100] direction, where an increase in epitaxially ordered atoms is evident. Potential energy analysis shows that the surface atoms in the layers have much higher potential energies than those inside the layer. This suggests that the bridging layer is mostly made up of a low-potential-energy core surrounded by more active surface atoms, which may be the key to fast bridging growth. The unique mechanism of cluster-assisted deposition enables clusters to deform and move as a cohesive unit, leading to rapid epitaxial-bridging growth. Therefore, when deposited on a porous or patterned substrate, there is potential to extend cluster-assisted mesoplasma CVD applications, such as layer transfer processes in industry.

### 3.5. Comparisons between the Simulation and the Experimental Results

In our previous research [5,37], we discussed the study of the high-rate epitaxial growth of pure silicon films using the mesoplasma CVD method. The experiments were conducted under various silane flow rates and RF power settings. We observed that the deposition rate significantly increased with rising RF power, and, at higher powers, the surface of the silicon films transitioned from an agglomerated structure to a planar structure with no visible grain boundaries. Meanwhile, in the current work, by varying the substrate temperature and cluster size, we found that the films formed by the impingement of smaller clusters had a smoother surface morphology, and the clusters could not sufficiently deform and spread at lower substrate temperatures, leading to globular shapes. The results demonstrated that substrate temperature and cluster size significantly influence the degree of cluster spreading and epitaxial growth of films.

We confirmed that increasing the RF power decreased the thickness of the thermal boundary layer during deposition, thus facilitating a smaller cluster size. Additionally, the substrate temperature would also increase with power. Therefore, our simulation results closely aligned with the experimental outcomes, providing deeper insights into the experimental results from an atomic level.

## 4. Conclusions

In this work, we studied the formation of mixed SiGe nanoclusters in the Si_0.5_Ge_0.5_ system and the impingement of SiGe nanoclusters on Si (100) smooth and trench surfaces using molecular dynamics simulations. To elucidate the rapid and uniform growth of SiGe/Si heterostructures at an atomic level, we analyzed the co-condensation process and cluster properties first, and then, by varying the substrate temperature and cluster size, we studied the evolution of the morphology, structure, and composition of the clusters after they impinged on the substrate surface.

Our results revealed that the Si and Ge atoms existed mainly in a vapor state, with small species at high temperatures. As the temperature decreased, significant cluster formation occurred, stabilizing at lower temperatures conducive to deposition environments. The clusters maintained a close SiGe ratio with the nominal value, remained amorphous, and displayed low potential energies in larger clusters due to a higher proportion of internal atoms. These properties, including thermal behaviors influenced by kinetic energies and atom interactions, suggest that SiGe nanoclusters are promising precursors for the deposition of epitaxial SiGe layers.

We also found that smaller clusters were better adapted to the substrate’s crystalline structure, resulting in a more uniform and smoother surface morphology for the films. In addition, higher substrate temperatures enhanced the diffusion and spreading of Si and Ge atoms across the substrate surface, promoting faster and more consistent layer formation. Moreover, the mole fraction of Ge in each layer closely matched the nominal fraction, indicating that the chemical uniformity of the layers had been achieved during fast epitaxial layer growth. The rapid epitaxial-bridging growth of the heterostructures during cluster impingement showed the distinctive features of the cluster-assisted deposition method as a versatile platform for synthesizing epitaxial films.

## Data Availability

The raw data supporting the conclusions of this article will be made available by the authors on request.
